# Thrombotic Microangiopathy in Haematopoietic Cell Transplantation: an Update

**DOI:** 10.4084/MJHID.2010.033

**Published:** 2010-11-03

**Authors:** Evi Stavrou, Hillard M. Lazarus

**Affiliations:** Department of Medicine, University Hospitals Case Medical Center, Case Western Reserve University, Cleveland, OH 44106

## Abstract

Allogeneic hematopoietic cell transplantation (HCT) represents a vital procedure for patients with various hematologic conditions. Despite advances in the field, HCT carries significant morbidity and mortality. A rare but potentially devastating complication is transplantation-associated thrombotic microangiopathy (TA-TMA). In contrast to idiopathic TTP, whose etiology is attributed to deficient activity of ADAMTS13, (a member of the A Disintegrin And Metalloprotease with Thrombospondin 1 repeats family of metalloproteases), patients with TA-TMA have > 5% ADAMTS13 activity. Pathophysiologic mechanisms associated with TA-TMA, include loss of endothelial cell integrity induced by intensive conditioning regimens, immunosuppressive therapy, irradiation, infections and graft-versus-host (GVHD) disease. The reported incidence of TA-TMA ranges from 0.5% to 75%, reflecting the difficulty of accurate diagnosis in these patients. Two different groups have proposed consensus definitions for TA-TMA, yet they fail to distinguish the primary syndrome from secondary causes such as infections or medication exposure. Despite treatment, mortality rate in TA-TMA ranges between 60% to 90%. The treatment strategies for TA-TMA remain challenging. Calcineurin inhibitors should be discontinued and replaced with alternative immunosuppressive agents. Daclizumab, a humanized monoclonal anti-CD25 antibody, has shown promising results in the treatment of TA-TMA. Rituximab or the addition of defibrotide, have been reported to induce remission in this patient population. In general, plasma exchange is not recommended.

## Introduction:

Allogeneic hematopoietic cell transplantation (HCT) is a useful therapeutic modality for a wide range of hematologic and non-hematologic conditions.^1^–^3^ Peripheral blood progenitor cell collection, the new ‘gold standard’ in hematopoietic cell harvesting, and non-myeloablative peripheral blood progenitor cell transplantation, have reduced treatment-related mortality and enabled an increasing number of patients with comorbid conditions as well as older patients to receive therapy for conditions such as acute leukemia, myelodysplastic syndrome, multiple myeloma and lymphoma. The obstacles to successful HCT include the development of acute and chronic graft-versus-host disease (GVHD), opportunistic infections, and other complications, one of which is transplantation-associated thrombotic microangiopathy (TA-TMA).^4^–^6^ The etiologies of this syndrome are diverse, and diagnosis of TA-TMA in this patient population requires a high degree of clinical suspicion. Moreover, management of TA-TMA remains a challenging task, mainly due to the poor response to therapeutic modalities that are beneficial in non-transplant-associated TMA.

## Pathologic and clinical features:

TMAs are defined by the association of microangiopathic hemolytic anemia, thrombocytopenia (platelet count < 100x10^9^/L) and ischemic manifestations related to the formation of platelet-rich thrombi in the microcirculation.^7^ TMAs include thrombotic thrombocytopenic purpura (TTP), and the hemolytic-uremic syndrome (HUS), and variants of these, which are characterized by ischemic manifestations involving the brain or gastrointestinal tract and/or kidneys, respectively.^8^ TMA may be primary, or occur secondary to other disorders such as pregnancy, infections, autoimmune diseases and the post-HCT state.^9^

The clinical presentation of TMA invariably includes the presence of schistocytes on the peripheral blood film and consumptive thrombocytopenia. Surrogate markers include DAT (direct antiglobulin test)-negative hemolytic anemia, an elevated serum lactate dehydrogenase (LDH), decreased serum haptoglobin and indirect hyperbilirubinemia. Coagulation studies are usually normal. A “pentad” of signs and symptoms was traditionally associated with classic TTP: thrombocytopenia, microangiopathic hemolytic anemia (MAHA), neurologic abnormalities, renal abnormalities and fever. This complete set of symptoms occurs in only 40% of patients, and more than 70% have only the triad of MAHA, thrombocytopenia, and neurologic changes at the time of diagnosis.^10^ In current clinical practice, thrombocytopenia, schistocytosis, and an elevated serum LDH in the appropriate clinical setting provide sufficient criteria for the diagnosis.^7^ The clinical manifestations of HUS are similar to TTP, although renal abnormalities, as opposed to neurologic dysfunction, often predominate.

Presentation of TA-TMA is similar to other forms of TMA; multiple contributing pathogenic factors have been implicated.^4^,^11^ These include endothelial cell injury due to toxic conditioning regimens (high-dose chemotherapy and total-body irradiation [TBI]), cytomegalovirus (CMV) infection, the use of calcineurin inhibitors such as cyclosporine, and a possible graft-versus-host effect on the endothelium.^4^,^12^–^14^ Because anemia, thrombocytopenia, renal impairment, and changes in mental status are common and may have multiple causes in the transplant population, diagnosis may be difficult.^15^ This observation currently is motivating experts in the field to reformulate a classification of TMAs more focused on pathophysiologic mechanisms rather than clinical symptoms.^16^, ^17^

## Diagnostic criteria:

Until recently, there were no widely accepted criteria for the definition of hematopoietic progenitor cell TA-TMA. The Blood and Marrow Transplant Clinical Trials Network (BMT CTN) and the International Working Group separately formed toxicity committees to develop a consensus formulation of criteria for diagnosing clinically significant TA-TMA; these are listed in Table 1.^18^,^19^

## Incidence and risk factors:

The reported proportion of patients developing a clinically significant TA-TMA syndrome has varied greatly. George and coworkers^15^ presented a review of published reports on TMA after allogeneic HCT. Twenty-eight different definitions of this syndrome have been used in the 35 reviewed reports. Reflecting the different definitions, the incidence of TA-TMA varied in these reports from 0.5 to 63.6% of HCT recipients, the median frequency of diagnosis being 7.9%. The mortality in the different series ranged from 0% to 100%; the overall mortality rate was 61%. Of the deceased patients, 35 autopsy reports were identified. Three of the autopsies attributed death to HUS due to observation of isolated renal TMA. The remaining 32 deaths were attributed to other causes, the most common being systemic infection, including CMV, Aspergillus species, adenovirus and human herpesvirus-6 (HHV-6). Eleven autopsies stated that there was no evidence of TTP-HUS.^15^ In another review, Pettitt and Clark^4^ estimated that TA-TMA occurs in 14% and 7% of allogeneic and autologous transplant recipients, respectively. A more recent report of more than 4000 HCT recipients estimated the frequency of severe TA-TMA to be 0.5% and 0.13% of allogeneic and autologous recipients, respectively.^6^ This varying incidence of TA-TMA among reported series likely reflects the level of physician awareness, the different diagnostic criteria, and the heterogeneity of the transplant population.

A variety of potential risk factors for the development of TA-TMA have been proposed (Table 2). Among the earliest reports, the use of cyclosporine (CsA) for the prevention of GVHD was recognized as a potential culprit.^20^,^21^ Intensive immunosuppression with other inhibitors of the Ca^2+^-activated phosphatase, calcineurin (tacrolimus),^22^–^24^ and (TBI)^25^ have been associated with TA-TMA as well. In the following section we examine the pathophysiology and clinical characteristics of both primary and secondary TMAs. The heterogeneity in clinical background (idiopathic TTP vs. disease-associated TMA, including TA-TMA) and plasma concentration of markers (normal vs. decreased ADAMTS13 activity) is well emphasized and represents a barrier to the development of clear treatment guidelines.

## Pathophysiology

### Idiopathic TTP:

Moake and colleagues^26^ were the first to describe the presence of very high molecular weight [so-called ultralarge (UL)] multimers of von Willebrand factor (vWF) in the plasma of a patient with recurrent TTP. Once released from stimulated endothelial cells, UL-vWF, not present in plasma from healthy individuals, promote excessive aggregation of platelets, primarily in the microvasculature. Some ULvWF multimers remain on the endothelial cell surface as long strings that adhere to platelets. The molecules responsible for the binding of UL-vWF to endothelial cells and platelets are believed to involve integrin αvβ3 and glycoprotein Ib (GpIb), respectively.^27^ Microvascular thrombosis and hemolytic anemia occur particularly in high shear stress locations, such as the microvasculature, that results in unfolding of ULvWF multimers and exposure of platelet binding sites.^7^,^28^

Moake hypothesized that a deficiency of a vWF cleaving protease might be responsible for the presence of ULvWF,^26^ but it was Furlan and colleagues^29^ et al., Tsai and Lian^30^ who first isolated a plasma metalloprotease that cleaved the peptide bond between the tyrosine 1605 and methionine 1606 in the central A2 domain of vWF. These investigators subsequently found a deficiency of this vWF-cleaving protease in a retrospective cohort of patients clinically diagnosed as having TTP.^29^,^30^ The protease was characterized in 2001 by Zheng and coworkers^31^ as a new (the thirteenth) member of the ADAMTS (A Disintegrin And Metalloprotease with Thrombospondin 1 repeats) family of metalloproteases and was thus called ADAMTS13.^16^,^31^–^34^ The primary role of ADAMTS13 is to regulate the multimeric structure of vWF by cleaving the most hemostatically active ULvWF multimers.^35^ Failure of this regulatory mechanism causes the highly adhesive, unusually large multimers of vWF to accumulate in plasma, which may lead to the microvascular thrombosis, tissue ischemia and infarction which are characteristic of TTP.

The estimated annual incidence of idiopathic TTP is 3.7 to 11 cases per million.^36^ In extremely rare cases, severe deficiency of ADAMTS13 (defined as <5% serum activity) is related to compound heterozygous or homozygous mutations of the ADAMTS13 gene (Upshaw-Shulman syndrome).^37^–^39^ Defects in coding of the metalloprotease gene, located on chromosome 9q34, result in functionally deficient enzyme. In the vast majority of cases, severe ADAMTS13 deficiency is secondary to the development of anti-ADAMTS13 autoantibodies that can be detected *in vitro*.^29^,^30^ Functional testing often is employed in which anti-ADAMTS13 autoantibodies are detected by their inhibitory effect on ADAMTS13 enzymatic activity.^40^ More recently, physical methods of detection have been used and identify either immunoglobulin G (IgG) or IgM species via enzyme-linked immunosorbent assay (ELISA).^41^,^42^ In more than 80% of acquired TTP, anti-ADAMTS13 antibodies are inhibitory IgG.^29^,^30^ Less frequently, the mechanism for acquired TTP may be different, involving anti-ADAMTS13 noninhibitory IgG or IgM^41^,^42^ autoantibodies that promote the clearance of ADAMTS13 from blood without inhibiting its activity.^41^

Adults with acquired idiopathic TTP require daily plasma exchange until neurologic symptoms have resolved and both a normal serum LDH and platelet count have been maintained for at least 2 to 3 days.^43^–^46^ Plasma exchange is thought to remove antibodies against ADAMTS13, while replacing the deficient protease.^47^,^48^ Plasma exchange results in the remission of TTP, which is usually fatal when untreated, in approximately 80%–90% of cases,^49^–^52^ generally without persistent organ damage. Production of ADAMTS13 autoantibodies may also be suppressed by high-dose corticosteroid treatment,^52^ although there is very little controlled data that demonstrates efficacy of steroids in the treatment of idiopathic TTP. Other therapies include use of the monoclonal antibody rituximab that is directed against the CD20 epitope on B lymphocytes; given weekly for 4–8 weeks, this agent will eliminate antibody-producing cells^53^–^57^ and has been shown to induce remission in refractory TTP, and to reduce the otherwise high incidence of relapse in these patients.^56^,^58^ Finally, splenectomy also has been shown to be effective in anecdotal cases of refractory TTP, and to reduce the incidence of relapse in small series.

### Hemolytic-uremic syndrome (HUS):

HUS refers to TMA that primarily affects the kidney, often causing oliguric or anuric renal failure.^59^ HUS may present with a variety of manifestations, with one variant occurring after Escherichia coli O157:H7 gastroenteritis, primarily in children.^60^ In adults, however, HUS occurs most commonly in association with pregnancy, with more than 90% of cases developing in the postpartum period.^61^ The characteristic histologic lesion of HUS consists of vessel wall thickening, with swelling and detachment of the endothelial cells from the basement membrane. In HUS, microthrombi are rich in fibrin and contain relatively little vWF.^62^ Their location is confined primarily to the kidneys and thus, renal failure is the dominant feature. HUS associated with renal failure in the absence of a diarrheal illness or other predisposing condition is commonly referred to as atypical HUS. It has been proposed that this group may, in part, consist of patients with complement system dysfunction owing to either a mutation of a complement-regulatory protein,^63^ an antibody directed at one of these proteins,^64^ or an activating mutation of a complement protein such as C3. Study of families with a history of HUS has implicated mutations in several proteins responsible for regulating the alternative complement pathway, namely complement factor H,^65^ membrane cofactor protein (MCP), and factor I (IF),^66^ as well as thrombomodulin.^67^ Exposure to agents potentially toxic to the vascular endothelium (such as certain viruses, bacteria, toxins, immunocomplexes, and cytotoxic drugs) may initiate local intravascular thrombosis.^68^ This action promotes C3bBb convertase formation and complement deposition within capillary vessels. Under normal conditions, however, factor H may effectively limit complement deposition and further extension of the process by modulating C3bBb activity.^69^ In contrast, when the factor H bioavailability is reduced due to decreased activity or is congenitally defective, C3bBb convertase formation and complement deposition may become uncontrolled. As a result, the microangiopathic process is extended, leading to full-blown manifestations of the disease.

### Secondary TMA:

Secondary forms of TMA refer to a diverse group of disorders with frequently overlapping clinical features (Table 3). An alternative classification that takes into consideration the underlying pathophysiologic mechanisms (immune-mediated vs. nonimmune-mediated) is presented here (Table 4). Immune-mediated forms result from autoantibodies against ADAMTS13.^41^,^42^,^70^ The second category (nonimmune-mediated) occurs from massive endothelial cell stimulation with consequent release of ULvWF multimers in amounts exceeding the system’s degradative ability, despite the presence of normal or only mildly reduced concentrations of ADAMTS13.^71^ Distinction between these forms should limit diagnostic uncertainty and assist with management strategies, namely implementation of plasma exchange and use of immunosuppressive therapy.

The most common physiologic condition present in the immune-mediated forms, which is often associated with severe ADAMTS13 deficiency, is pregnancy.^29^,^36^,^72^,^73^ Immune-mediated TMA in pregnancy should be distinguished from a number of pregnancy-associated TMAs such as preeclampsia, hemolysis with elevated liver enzymes and low platelets (HELLP) syndrome, acute fatty liver of pregnancy, and antiphospholipid syndrome.^74^,^75^ Accurate diagnosis is essential since plasma exchange is indicated for pregnancy-associated TTP while fetal-placental delivery is therapeutic for HELLP syndrome.

The association between TTP and SLE has been well recognized in clinical and histologic reports.^76^ Severe deficiency of ADAMTS13 activity is predominantly associated with the presence of inhibitory anti-ADAMTS13 IgG.

ADAMTS13 levels are not generally decreased with infections such as HIV, suggesting an alternative mechanism for TMA in these patients.^77^,^78^ Cases of TMA associated with severe ADAMTS13 deficiency and inhibitory anti-ADAMTS13 IgG have been reported with influenza A,^79^ legionella pneumonia^80^ and brucellosis.^81^

Antibodies that inhibit plasma ADAMTS13 have also been demonstrated in patients with ticlopidine^82^ or clopidogrel-associated TMA.^83^ The immune dysregulation by these thienopyridine compounds might be analogous to the anti-RBC antibody ‘escape’, associated with the antihypertensive medication α-methyldopa.^84^

The most frequent concomitant conditions associated with TMA forms presenting with normal or mildly reduced levels of ADAMTS13 (greater than 10% serum activity) are malignant hypertension,^17^ metastatic tumors,^85^ solid organ transplantation, HCT (particularly allogeneic transplants), and the use of drugs such as cyclosporine, mitomycin, and α-interferon.^34^

Despite having some features in common, TA-TMA differs from *de novo* TTP in many aspects including the absence of severe ADAMTS13 deficiency, a different spectrum of clinical symptoms, poor response to plasma exchange, and the lack of evidence of systemic microthrombus formation.^15^ Several small retrospective studies of TA-TMA encompassing a total of 33 HCT recipients also suggest that severe ADAMTS13 deficiency may be rare among this patient population.^86^–^88^ Indeed, other prospective reports in HCT recipients suggest that the majority of these patients experience only a mild decrease in ADAMTS13 activity (usually after the cytotoxic conditioning) that can persist for weeks; however, severe ADAMTS13 deficiency was rare.^89^,^90^ These data suggest that, unlike idiopathic TTP, ADAMTS13 deficiency is not the primary component of the pathophysiology of TA-TMA and other factors may play a more central role.

### Role of endothelial cell injury:

For a long time, many authorities have considered endothelial cell injury as the central and likely inciting factor that sustains the microangiopathic process in TMA, including the post-transplantation state. As early as 1942, Altschule^91^ suggested that microvascular endothelial cell activation was the primary event causing platelet deposition in arterioles and capillaries with secondary “clearance of enormous numbers of platelets from the circulation”. Endothelial cells synthesize many substances involved in coagulation and fibrinolysis including vWF, thrombomodulin, tissue-type plasminogen activator (tPA), plasminogen activator inhibitor (PAI-1), protein S, prostacyclin (PGI2), and nitrous oxide (NO). Alterations in the concentration of these substances have been reported in idiopathic TTP and TA-TMA.^92^,^93^ Whether these alterations represent an initiating effect or simply are reflective of endothelial cell injury, remains elusive. Levels of vWF antigen and soluble thrombomodulin were measured in patients with idiopathic TTP and TA-TMA.^94^ vWF antigen and thrombomodulin levels were elevated in both patient groups compared to controls. Thrombomodulin concentrations were significantly higher in TA-TMA compared to idiopathic TTP, supporting a role for endothelial cell damage in the former. Gordon and colleagues^95^ demonstrated that protein C deficiency correlated with thrombotic complications in patients undergoing HCT. Several groups have reported elevated plasma levels of fibrinogen, tPA, PAI-1, vWF antigen, thrombomodulin, and intercellular adhesion molecule 1 (ICAM-1).^96^–^100^ Kanamori et al.^100^ proposed that measurement of thrombomodulin levels on day 14 post-HCT may be useful in surveillance for TA-TMA. Cohen and colleagues^93^ proposed that endothelial cell injury is pathognomonic of TA-TMA as well after they demonstrated absent endothelial cell PGI_2_ release and scanning electron microscopy (EM) evidence of endothelial cell damage with TMA after allogeneic HCT.

In 1996, Laurence and co-workers^101^ demonstrated that plasma from four acute TTP patients could induce apoptosis of cultured endothelial cells of microvascular but not of large-vessel origin. Ultrastructural changes became apparent within 30 minutes after exposure of endothelial cells to TTP sera and fresh complement; virtually every cell quickly developed numerous cytoplasmic inclusions, followed by complete cytoplasmic and nuclear degeneration. Apoptosis was independent of tumor necrosis factor-α (TNF-α) or the presence of CD36 on microvascular endothelial cells, but was linked to the rapid induction effect of Fas (CD95) on these cells. Use of soluble anti-Fas antibodies suppressed the endothelial cell apoptosis mediated by TTP plasma.^101^

The presence of circulating endothelial cells (CEC) recently has been recognized as a useful marker of vascular damage. Usually absent in the blood of healthy individuals, CEC counts are elevated in diseases marked by the presence of vascular insult.^102^ Recently, another endothelial cell marker linked with vascular dysfunction has been identified. Endothelial cell microparticles (EMP) are vesicles formed by the endothelial cell membrane after injury or activation. The phenotypic profile of EMP can vary considerably, depending on whether parent cells have undergone either activation (abundant CD62E^+^) or apoptosis (predominantly CD31^+^).^103^ Microparticle (MP) formation has been demonstrated using *in vitro* endothelial cell activation by cytokines such as TNF-α and interleukin (IL)-1.^104^ It has also been demonstrated that EMP have procoagulant activity, defined by platelet factor 3 activity and tissue factor (TF).^105^ In prothrombotic states, Shet et al.^106^ reported raised tissue-factor (TF)-positive EMP in patients compared with controls and a strong correlation with procoagulant activity. Increased production of EMP has been demonstrated in idiopathic TTP.^107^ There are few data concerning the release of EMP in the course of HCT exist; however, a recent report suggests that EMP increase in the setting of acute GVHD, but not immediately after non-myeloablative conditioning regimens.^108^ Increases in circulating platelet- and monocyte-derived MP have also been observed in the post-transplantation period, including in one case of TA-TMA.^109^

In the HCT setting, proposed mechanisms of endothelial cell injury include toxic conditioning regimens with high-dose chemotherapy,^110^–^114^ TBI,^115^ infections (CMV, HHV-6),^116^,^117^ and GVHD.^96^,^98^,^117^,^118^ Evidence of TA-TMA was found in one analysis of allogeneic HCT recipients receiving cyclosporine as GVHD prophylaxis but not in those treated with methotrexate (MTX).^14^ In addition, other calcineurin inhibitors (e.g., tacrolimus) used for GVHD prophylaxis have been associated with increased production of thromboxane (TX) A_2_ and decreased production of PGI_2_.^119^,^120^ Addition of sirolimus to a calcineurin inhibitor has been associated with potentiation of these effects.^121^ Data from renal transplant recipients developing TA-TMA on an immunosuppressive regimen of cyclosporine and sirolimus suggest that the combination of these agents concomitantly targets the molecular control of cell death and repair at the EC level.^121^ The end result is loss of endothelial cell integrity, and generation of a proinflammatory, procoagulant state likely leading to predilection for secondary TMA.

### Risk factors and prognosis:

Non-modifiable risk factors for development of TA-TMA include female gender, African American race, and older age.^36^,^122^,^123^ Prior medical history of severe hepatic dysfunction and advanced primary disease also increase the risk of developing TA-TMA.^124^,^125^ Treatment-related risk factors include: unrelated donor transplants;^88^,^122^–^124^,^126^–^128^ HLA-mismatched donors;^124^ fludarabine-based non-myeloablative conditioning regimens;^88^,^129^ busulfan and TBI myeloablative conditioning.^25^,^123^,^130^ The incidence of TA-TMA did not differ according to graft source, e.g. bone marrow versus peripheral blood.^122^ As mentioned above, use of calcineurin inhibitors such as cyclosporine,^14^,^126^,^131^–^133^ tacrolimus,^24^,^130^,^132^ and sirolimus^132^,^134^,^135^ are also associated with the development of TA-TMA. Infections and the development of GVHD also increase the risk of developing TA-TMA.^14^,^21^,^123^,^124^,^128^ In the HCT patients, non-transplantation etiologies of TMA such as idiopathic TTP and HUS should always be considered in the differential diagnosis, as they may coexist with the primary hematologic disease. In TA-TMA, poor prognostic indicators include: patient age > 18 years, a graft source from an unrelated or haploidentical donor,^123^ at least five schistocytes per high-power field on peripheral film,^136^ TA-TMA in the absence of sirolimus,^134^ and nephropathy.^122^ Specifically, Uderzo and coworkers^123^ reported three factors statistically significant in predicting outcome of TA-TMA: adult age, unrelated or haploidentical graft source, and high TMA index (elevated LDH-platelet ratio). A retrospective cohort analysis of myeloablative allogeneic HCT recipients showed that sirolimus exposure constitutes a risk factor for the development of TA-TMA (10.8% in the sirolimus-exposed subjects vs. 4.2% in the non-sirolimus group)^134^ but is also a favorable prognostic indicator in terms of TA-TMA overall survival (58.3% for TA-TMA related to sirolimus exposure vs. 11.1% in the non-sirolimus group)^134^ and renal recovery (92% vs. 78% respectively).^134^ Martinez and colleagues^136^ reported lower one-year survival in patients with TA-TMA than in patients without TA-TMA (27 ± 18.1% for TA-TMA with high schistocyte counts; 53 ± 15% for TA-TMA with low schistocyte counts; vs. 78 ± 7% in patients without TA-TMA, p< 0.0001). A survey of the European Group for Blood and Marrow Transplantation (EBMT)^122^ conducted among forty-five centers included 406 patients transplanted, and reported an incidence of TA-TMA of 6.7%. The only factor predictive of resolution of TA-TMA was the absence of nephropathy.^122^

## Therapeutic modalities

At the present time there is no consensus on what constitutes appropriate therapy for patients with TA-TMA. Initial attempts should focus on the following: i) eliminating possible causative conditions such as treating underlying infections and controlling acute GVHD; and ii) pharmacologic therapy with medications such as daclizumab, defibrotide and rituximab. The rationale for use of these agents is based on empirical benefit

### Eliminating Risk Factors and Consideration of Plasma Exchange:

Cyclosporine, tacrolimus and sirolimus should be discontinued immediately and replaced with alternative immunosuppressive medications. Corticosteroids, mycophenolate mofetil, azathioprine and methotrexate can be used as appropriate alternatives. Withdrawal of cyclosporine with initiation of plasma exchange/apheresis has shown response rates of up to 63%.^137^ In all reported cases cyclosporine was discontinued at the time of diagnosis of TA-TMA, and the effect of this intervention in isolation cannot be determined as patients went on to have therapeutic plasma exchange.

Unlike the situation in idiopathic TTP, responses to plasma exchange alone are suboptimal in TA-TMA. Reported response rates vary between 0–49%,^138^,^139^ compared with 78–91% in patients with idiopathic TTP.^45^,^140^ Further, rise in platelet count, the usual marker for response to plasma exchange, cannot be relied upon in TA-TMA because platelet engraftment may not yet have occurred. In addition, plasma exchange procedures are associated with a significant number of complications, including systemic infections, catheter thrombosis, bleeding, pneumothorax, pericardial tamponade, and with plasma infusion, serum sickness and anaphylaxis.

Based on the incomplete responses and high complication rates, we do not advocate use of this procedure but rather alternative therapeutic approaches as discussed below.

### Daclizumab:

Daclizumab is a humanized monoclonal anti-CD25 antibody, which targets the chain of the IL-2 receptor.^141^ This agent is 90% humanized, retaining only 10% of the original murine compartments in the critical hypervariable segments for binding specificity. Daclizumab has been used to decrease the incidence of acute rejection in solid organ transplants including renal,^142^ hepatic, cardiac,^143^,^144^ and lung transplantation.^143^,^145^ Daclizumab also has been used successfully in T-cell mediated autoimmune diseases such as multiple sclerosis,^146^–^148^ pure red cell aplasia,^149^ and aplastic anemia.^150^

In the HCT setting, intravenous daclizumab (with a serum half-life of 20 days) has been used to prevent or treat acute GVHD;^151^ more recently, this agent has been used for the treatment of TA-TMA. Adverse effects include an increased risk for bacterial, candida and aspergillosis infections, as well as CMV reactivation. Through its effect on depleting alloreactive T-cells, daclizumab can substitute for a calcineurin inhibitor. Wolff et al.^132^ used daclizumab at an initial loading dose of 2mg/kg and then 1mg/kg weekly. Nine of 13 affected patients attained complete remission after therapy.^132^ Four of the patients who had a complete remission from TMA also had complete resolution of active GVHD. A fifth, complete remitter patient from both TMA and GVHD died of primary disease relapse; the remaining eight patients died from infections, GVHD or multiorgan failure.^132^ The long half-life and potent immunosuppressive effect make this agent a promising treatment modality that merits further investigation.

### Defibrotide:

Defibrotide is a large, single-stranded polydeoxyribonucleotide, derived from porcine mucosa by controlled depolymerization. It has been found to have potent anti-thrombotic, anti-ischemic, anti-inflammatory, and thrombolytic properties, without significant systemic anticoagulant effects.^127^,^152^–^158^ This drug exerts its properties by inhibition of TNFα-mediated endothelial cell apoptosis *in vitro*,^148^ decreasing the activity of PAI-1 and increasing endogenous tissue plasminogen activator (tPA) function.^159^

Hepatic veno-occlusive disease (VOD) is a potentially lethal complication of both allogeneic and autologous HCT,^156^,^160^,^161^ especially after prior exposure to the immunoconjugate gemtuzumab ozogamicin.^162^ In some studies, the incidence of hepatic VOD after HCT approaches 20% with mortality ranging from 7% to 50%.^163^ The pathogenesis of VOD involves injury to the sinusoidal endothelial cells, leading to occlusion of small vessels with fibrin deposition and disruption of hepatic function. Previous attempts at therapy using either heparin or tPA have been unsuccessful.^164^,^165^ Defibrotide therapy has improved outcomes for hepatic VOD that develops after HCT (30% to 60% CR rate).^166^–^170^ Given the similarities in pathophysiology with TA-TMA, including loss of small vessel endothelial cell integrity, Corti and coworkers^155^ reported that 3 of 12 affected patients with TA-TMA given oral defibrotide achieved partial remission, while 5 of 12 patients achieved a complete response.^155^ Because the effects of defibrotide are exerted locally within the vascular bed, it is usually well tolerated with no significant systemic effects on coagulation such as seen during treatment with tPA.

### Rituximab:

Rituximab, discussed above, has been used with increasing frequency for the treatment of various hematologic and rheumatologic disorders including idiopathic TTP,^171^–^173^ acquired coagulation factor inhibitors,^174^,^175^ antiphospholipid antibody syndrome,^173^ systemic lupus erythematosus^176^ and rheumatoid arthritis.^177^,^178^ Rituximab use in relapsed, refractory TTP is linked to its ability to eliminate antibodies to ADAMTS13.^171^ Au et al.^179^ treated five TA-TMA patients refractory to a week of plasma exchange and prednisolone with rituximab 375mg/m^2^/week for four doses. Four attained complete remission; two patients recovered after receiving two weeks of rituximab.^179^ At a median follow-up of 305 days, 3 of 4 responders remained in remission^179^ but the fourth responder died of sepsis. The only non-responder died of multi-organ failure after three weeks.^179^ ADAMTS13 antigen levels were marginal or low either post-HCT or at the onset of TMA and did not change significantly after rituximab-induced remission.^179^ It remains unclear whether these patients actually had TA-TMA (or TTP), thus making the use of rituximab in this setting uncertain. In addition, there are no established guidelines for recommending duration of rituximab treatment. Given the lack of reliable markers for remission (serum ADAMTS13 activity and anti-ADAMTS13 antibody levels), maintenance rituximab therapy cannot be recommended.

### Other modalities and future directions:

Single agent response rates for the antiplatelet agents aspirin and dipyridamole approximate 10%,^49^,^180^ a result indistinguishable from the natural history of TTP. Antiplatelet agents have not been convincingly shown to increase the response to plasma exchange^45^,^51^,^181^ and may promote bleeding in the setting of acute thrombocytopenia and invasive procedures.^51^,^182^ Hence, their use as first-line treatment of TMA, including TA-TMA, cannot be recommended. Although not verified *in vivo*, intravenous immunoglobulin (IVIG) has been used therapeutically based on a report that IgG from healthy individuals inhibits the capacity of TTP plasma to agglutinate platelets *in vitro*.^183^ A recent study described a response to the combination of plasma exchange and IVIG in a patient who was refractory to plasma exchange alone.^184^ There are anecdotal reports of favorable responses of TTP to vincristine,^180^,^185^,^186^ as well as other immunosuppressive therapies such as azathioprine, cyclophosphamid, and staphylococcal protein A immunoadsorption.^187^,^188^ By analogy, there are case series of use of these agents in the setting of TA-TMA. Results were disappointing and difficult to interpret since all of the patients received concurrent therapy with plasma exchange.^128^,^139^,^189^

On-going studies for further investigation of the pathogenesis of TA-TMA involve medications that modulate the endothelial cell inflammatory response.^190^ These agents include statins^190^ and bosentan,^191^ an endothelin receptor antagonist with protective effects in *in vivo* ischemia-reperfusion injury models. Anti-oxidant agents, such as nitric oxide donors which limit vascular injury caused by free-radicals, also may alter the course of the disease.

## Conclusions:

TA-TMA is an uncommon but devastating complication of HCT. Evidence suggests that it represents the final common pathway of multiple, frequently confounding variables such as conditioning regimens, use of calcineurin inhibitors, acute GVHD and opportunistic infections. The elevated blood concentrations of vWF antigen confirm endothelial cell injury. The typically incomplete responses and high mortality rates call for better therapeutic approaches. An alternative classification that takes into consideration the underlying pathophysiologic mechanisms is presented here and should limit diagnostic uncertainty. Accurate diagnosis is instrumental in designing future studies comparing management strategies and outcomes among different series. Treatment of TA-TMA consists of discontinuing offending agents and substituting calcineurin inhibitors with daclizumab or other immunosuppressives. Due to questionable efficacy and significant associated adverse events, plasma exchange, in general, is not recommended. Finally, monitoring production of endothelial cell microparticles or protein concentration changes of vWF, soluble thrombomodulin, and PAI-1, which occur in the setting of endothelial cell injury, may be useful in detecting early onset of TA-TMA. In line with these considerations, interventions directed at improving endothelial cell function, accelerating endothelial cell recovery from injury and preventing apoptosis of these cells are potential goals for future developmental therapies.

## Figures and Tables

**Table 1:** Diagnostic criteria for transplantation-associated TMA

**Blood and Marrow Transplant Clinical Trials Network (BMT CTN) toxicity committee consensus definition for TMA^18^**	**International Working Group Definition for TMA^19^** **All of the following are present:**
1) RBC fragmentation and ≥ 2 schistocytes per high-power field on peripheral film	1) Increased percentage (> 4%) of schistocytes in the blood
2) Concurrent increased serum LDH above institutional baseline	2) *De novo*, prolonged or progressive thrombocytopenia (platelet count less than 5 × 109/l or a 50% or greater decrease from previous counts)
3) Concurrent renal^a^ and/or neurologic dysfunction without other explanations	3) Sudden and persistent increase in LDH
4) Negative direct and indirect Coomb’s test results	4) Decrease in hemoglobin concentration or increased red blood cell transfusion requirement
	5) Decrease in serum haptoglobin concentration

Abbreviations: LDH: lactate dehydrogenase; TMA: thrombotic microangiopathy; TA-TMA: transplantation-associated thrombotic microangiopathy.

a Doubling of serum creatinine from baseline (baseline=creatinine before hydration and conditioning) or 50% decrease in creatinine clearance from baseline.

**Table 2:** Potential risk factors for development of TA-TMA

**Risk factor**	**References**
Female gender	21, 36, 122, 123
Older age	21, 36, 123
African American race	36
Advanced primary disease	124
Unrelated donor transplants	122–124, 126–128
HLA-mismatch (one or more loci)	124
Nonmyeloablative conditioning regimens (fludarabine-based regimens)	88, 129
High-dose busulfan (16mg/kg)	130
Total body irradiation	25, 123
Cyclosporine	14, 126, 131–133
Tacrolimus	24, 130, 132
Cyclosporine and sirolimus	134, 135
Infections	124, 128
Acute GVHD	14, 21, 123, 124, 126, 128

Abbreviations: TA-TMA: transplantation-associated thrombotic microangiopathy; GVHD: graft-versus-host disease; HLA: human leukocyte antigen.

**Table 3:** Primary and secondary thrombotic microangiopathies

**Primary TMA**	**Secondary TMA**
- Hereditary TTP - Idiopathic TTP	Immune-mediated - Pregnancy - Autoimmune disorders - Infections - Medications (clopidogrel, ticlopidine)
- Hereditary (atypical) HUS - Sporadic (Shigatoxin-associated)	Non-immune mediated - Malignant hypertension - Solid organ transplantation - HCT - Metastatic tumors - Medications (cyclosporine, tacrolimus, IFN-α, Mitomycin C)

Abbreviations: TMA: thrombotic microangiopathy; TTP: thrombotic thrombocytopenic purpura; HUS: hemolytic uremic syndrome; HCT: hematopoietic cell transplantation; IFN-α: interferon alpha.

**Table 4:** Pathophysiologic classification of primary and secondary thrombotic microangiopathies.

**Immune Mediated**	**Non Immune Mediated**
Primary Idiopathic TTP Atypical HUS secondary to inhibitory antibodies to complement – regulating proteins	Primary Hereditary TTP Hereditary atypical HUS secondary to mutations in complement – regulating proteins
Secondary Pregnancy Autoimmune disorders Infections Medications (clopidogrel, ticlopidine)	Secondary Malignant hypertension Solid organ transplantation HCT Metastatic tumors Medications (cyclosporine, tacrolimus, IFN-α, Mitomycin C)

Abbreviations: TMA: thrombotic microangiopathy; TTP: thrombotic thrombocytopenic purpura; HUS: hemolytic uremic syndrome; HCT: hematopoietic cell transplantation; IFN-α: interferon alpha.
